# Maternal contributions to individual behavior in *Daphnia*

**DOI:** 10.1093/plankt/fbag021

**Published:** 2026-04-07

**Authors:** Andrzej Mikulski, Joanna Pijanowska

**Affiliations:** Department of Hydrobiology, Institute of Ecology, Faculty of Biology, University of Warsaw, Żwirki & Wigury 101, Warsaw 02-089, Poland; Department of Hydrobiology, Institute of Ecology, Faculty of Biology, University of Warsaw, Żwirki & Wigury 101, Warsaw 02-089, Poland

**Keywords:** *Daphnia*, anti-predator defense, diel vertical migrations, maternal effect, DVM, phenotypic plasticity, Predation Risk Allocation Hypothesis

## Abstract

In a laboratory experiment conducted in thermally stratified glass tubes, so-called “plankton organs”, we tested two alternative hypotheses concerning the role of maternal effects on *Daphnia* vertical migration behavior induced by the presence of a fish predator. The first hypothesis states that maternal experience does not influence the induction of this behavior because it is quickly induced and does not require information about the persistence of the threat. The second hypothesis states that maternal fear increases the intensity of the individual responses of their descendants to the threat. However, both hypotheses turned out to be false. Fear experienced by the mother discourages offspring from incurring the costs of defense. The obtained results allow to hypothesize that the process of optimizing defense mechanisms may integrate decisions across several generations and include compensation for costs of defense in the previous generation. Regardless of the need to further validate this hypothesis, our results demonstrate the complexity of optimizing the induction of defense mechanisms in *Daphnia*.

## INTRODUCTION

Maternal effects (ME) are one of the key mechanisms generating phenotypic diversity in natural populations (Barnardo, 1996; Mousseau and Fox, 1998). The adaptive role of ME has been discussed in numerous reviews and book chapters (e.g. Badyaev and Uller, 2009; Inchausti and Ginzburg, 2009; Cameron and Garcia, 2013). A notable example of ME is the anticipatory maternal effect (for a review see Marshall and Uller, 2007), whereby the norm of offspring responses to environmental change is shaped by mothers, according to the anticipated selection landscape during their ontogeny.

*Daphnia*, a planktonic cladoceran known for its broad reaction norms, is a perfect model for research on phenotypic plasticity. This plasticity is revealed in response to various selection factors, including predator pressure (Gliwicz, 1994), the quantity and quality of available resources (Gliwicz, 1990; Gliwicz and Lampert, 1990; Gliwicz and Guisande, 1992), and pressure from abiotic factors (Gliwicz and Sieniawska, 1986). The complex responses of *Daphnia* to these factors, including morphological reconstruction (Dodson and Havel, 1988; Dodson, 1989), changes in life history (Riessen, 1999), and behavioral modifications (Dawidowicz and Loose, 1992), have been widely documented. Flexible responses in *Daphnia* to selection factors are modified by intergenerational transmission (Ellis *et al.,* 2020; Lee, 2025). In our previous studie (Mikulski and Pijanowska, 2017), we attempted to determine the impact of ME on individual fitness by analysing phenotypic traits under maternal and individual control and investigating the roles of mothers’ and individual perceptions of fish predation threat in shaping the *Daphnia* phenotype. The results suggested that the contribution of maternal and self-perception of risk to shaping the phenotype of an individual depends on the time required to express a specific trait. A long initiation time for trait change and a potentially long “return” time increase the importance of maternal risk perception in shaping the offspring phenotype.

If this is the case, the ME should not be involved in shaping features that can change immediately upon perception of an emerging threat and quickly return to their initial form once the risk has passed. The initiation of diurnal vertical migrations (DVM) in *Daphnia* meets this condition (Ahlers and von Elert, 2025). This behavior involves *Daphnia* remaining in the deep layers of the lake during the day, where they are inaccessible to visually oriented predators and moving to the warm epilimnionat night to safely access food resources and grow quickly (Lampert, 1989; Gliwicz, 2003). On the other hand, *Daphnia* response to the fish kairomone is rapid, but not immediate. A significant effect occurs approximately two hours after the signal is perceived (De Meester and Cousyn, 1997). If the kairomone appears in the environment alongside actively feeding fish, *Daphnia* response may be delayed. Assuming that the ME informs on the ongoing threat, it can stimulate individuals to accept greater defense costs and implement more far-reaching forms of defense, promoting permanent residence of *Daphnia* in deeper water layers.

Previous studies of ME on offspring behavioral traits in *Daphnia* have focused on responses to abiotic factors, especially to UV radiation. Offspring of mothers exposed to radiation exhibited significantly weaker behavioral responses to this stimulus (Sha and Hansson, 2022). This could be due to habituation, or to a physiological preparation of the offspring to UVR. However, responses to biotic factors (including predation) may be founded on other mechanisms. Studies investigating the impact of maternal exposure to predatory threats on offspring behavior across various species has led to ambiguous results. In some cases, such exposure appears to have no effect on offspring behavior, as demonstrated in snails (Beaty *et al.,* 2016) and spider mites (Freinschlag and Schausberger, 2016). However, it can also induce or enhance defense mechanisms in the offspring of crickets (Storm and Lima, 2010), snails (Luquet and Tariel, 2016) and sticklebacks (Stein *et al.,* 2018). Moreover, other studies have shown that maternal exposure to predatory threats can also inhibit defense mechanisms in the offspring of other snail species (Donelan and Trussell, 2015).

None of the cited studies examined the influence of maternal exposure on offspring DVM behavior. Therefore, their results cannot be used to make predictions about the possible impact of ME on migratory behavior of their offspring under predation threat. Therefore, our assumptions are based on theoretical predictions of the potential adaptive consequences of the ME in the induction of DVM migration. Our study aims to test two hypotheses emerging from these considerations. First we hypothesized that a ME is not involved in inducing vertical migration in *Daphnia*, as relatively fast inducible defense. The second is that ME promote the permanent residence of *Daphnia* in deeper water layers, despite the associated additional costs.

## METHODS

In the experiment, we used *Daphnia magna* from a clone originating from the shallow, coastal reservoir Binnensee in Germany (54°19′29.5″N 10°37′43.8″E), where these animals periodically coexist with fish. Clones from this lake are commonly used to study the defense mechanisms of cladocerans in response to the presence of planktivorous fish, including vertical migrations (Lampert, 1991). This clone was established from ephippial eggs and has earlier been used in our studies on ME (e.g. Mikulski, 2001; Mikulski and Pijanowska, 2017). Also, it was earlier recognized as strongly responding to the presence of fish threat by performing various defensive strategies, including DVM.

The animals were bred for three generations in constant dim light at a temperature of 22°C. They were kept separately in 200 mL glass containers filled with aged lake water filtered through a 0.2 μm membrane filter. They were fed green algae *Acutodesmus obliquus* at a concentration of 1 mg C_org_·L^−1^. The algae-containing water was changed daily. Subsequent experimental generations originated from the offspring of the first clutch of previous generation.

The neonates from the first clutch of the third generation of females were split randomly between ten 0.9 L jars no later than six hours after birth, with ten individuals in each. This initiated culturing the maternal generation. Half of the jars were filled with the medium used so far; the other five jars were filled with the same medium with the addition of crucian carp kairomone obtained according to the recipe of von Elert and Loose (1996), at a concentration corresponding to one fish in 10 L of medium. According to this method, the kairomone was enriched from water in which the fish have been kept, using solid-phase extraction (SPE) on non-polar C18 phenyl SPE cartridges (500 mg, Analytichem Int.). After cleaning the cartridge with ultrapure water, the kairomone was eluted using methanol and concentrated in an evaporator. Such obtained kairomone solution retained its activity for many months.

**Fig. 1:**
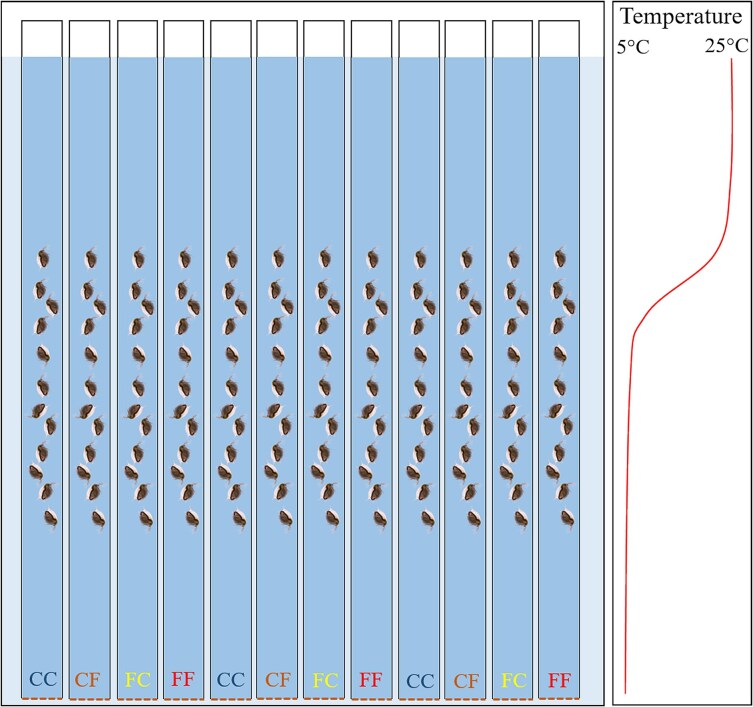
Schematic illustration of the experimental setup; the letters at the bottom of the tubes indicate the different variants of the experiment: *Daphnia magna* directly exposed (CF-orange, FF-red) or not exposed (CC-blue, FC-yellow) to fish kairomone, with their mothers exposed (FC-yellow, FF-red) or not exposed (CC-blue, CF-orange) to kairomone.

The offspring of the first clutch of females that were cultured in jars were separated from their mothers, and transferred to the so-called "planktonic organs" (Dawidowicz and Loose, 1992), which were 600 mm long glass tubes with an internal diameter of 8 mm and a bottom made of permeable fabric of 400 μm mesh size, placed vertically in a water-filled aquarium (see Fig. 1). Animals from mothers that were and were not exposed to the presence of the kairomone were alternately placed in subsequent tubes, 12 *Daphnia* in each of the twelve tubes—three tubes for each of the four experimental variants. Finally, there were three tubes (replicates) containing twelve individuals (pseudoreplicates) in each treatment. At the beginning of the experiment, the food concentration in the tubes was uniform throughout the water column at 1 mg C_org_·L^−1^. After the animals were placed in the experimental setup, the temperature regulation system was activated, creating strong thermal stratification in the tubes (25°C at the surface, 5°C at the bottom, with a sharp thermocline at half depth). The aquarium containing the tubes was illuminated from above. The light intensity at the surface was 46 μmol·cm^−2^·s^−1^ and at the bottom it was 28 μmol·cm^−2^·s^−1^. A 12 L:12D photoperiod was established. The next day, at noon, the depths selected by each of the 144 animals were recorded. Then, a medium containing algae at a concentration of 2 mg C_org_·L^−1^ and at a temperature of 25°C was poured from the top into each of the tubes. The concentration of 2 mg C_org_·L^−1^ in half the tube's volume was quantitatively equivalent to the concentration of 1 mg C_org_·L^−1^ in the whole tube, i.e. the concentration that had been used previously. The medium poured into half of the tubes contained fish kairomone and, thus, four variants of the experiment have been created. They differed in whether or not *Daphnia* were exposed to kairomones in the maternal generation and the current presence of kairomones. The amount of medium poured in filled the upper half of the tube, pushed an equal volume of water out of the lower part. Consequently, the upper layer in all tubes contained fresh medium with an algal concentration of 2 mg C_org_·L^−1^, and in half of the treatments, it also contained fresh kairomone. The lower portion still contained an old medium deprived of algae consumed by *Daphnia*. In half of tubes, it also contained degraded kairomone. After 180 minutes later, the depth at which the animals resided was recorded. As kairomone decomposes rapidly (almost 90% of kairomone biodegrades in 24 hours—see Ahlers and von Elert, 2025), we assumed that there were no reaction-inducing concentrations of kairomone in the experimental system prior to the daily water change, and that the daily supply represented a fresh information about the hazard. Recording of the depth of residence and water change procedures described above were repeated for five consecutive days. After the final recording, the animals were removed from the tubes, photographed and measured alive with the MultiScan system, with 1 μm accuracy.

The effects of maternal exposure to the presence of kairomone, direct exposure to the kairomone, *Daphnia* age (i.e. experimental day), and effect of daily addition of fresh medium with or without kairomone on the depth at which the animals resided were examined using Linear Mixed Effects Model—LMM with individual depth records as pseudoreplicates nested within each tube, considered to be true replicates. Tube effects (replicates) was random and other tested effects were fixed. A replication x day random effect was also included to account for variability between observations in the same tube on a given day. Differences in the depth of *Daphnia* residence between different treatments and before vs after medium change were analysed using Tukey tests with correction for multiple comparisons. The comparison of the depth of animals in different treatment groups include the depths before and after the medium change, accurately reflecting the average daily difference between groups. All statistics were performed using the RStudio package version 2025.09.0 Build 387, R version 4.5.1.

As the data on *Daphnia* size did not meet the criteria for parametric analyses, treatment effects on size were analysed using the non-parametric Kruskal–Wallis test and Mann–Whitney test as post-hoc in Statistica 6, StatSoft Inc.

## RESULTS

**Fig. 2:**
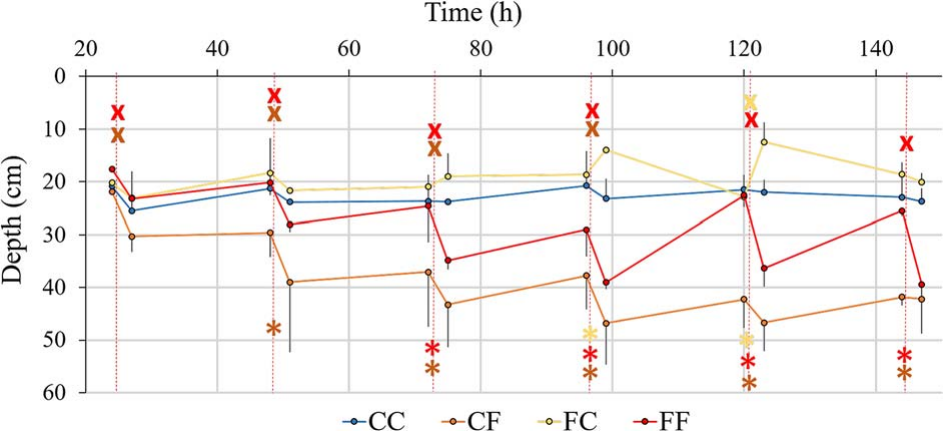
The day depth of residence of *D. magna* directly exposed (CF, FF) or not exposed (CC, FC) to fish kairomone, with their mothers exposed (FC, FF) or not exposed (CC, CF) to kairomone—average ± 1SD; the dashed vertical line indicates the moment at which fresh medium with or without kairomone (according to the treatment) was added; asterisks indicate significant differences in the depth selected by *Daphnia* compared to the control group (CC, unexposed animals from unexposed mothers); x symbols indicates significant changes between the depths selected by *Daphnia* before and after change of medium (adding fresh food to all and fresh kairomone to appropriate treatments); *P* < 0.05, Tukey’s post-hoc test.

The vertical distribution of *Daphnia* was influenced by all the studied factors (Fig. 2, Table I). The strongest and most consistent migration behavior was observed in individuals whose mothers were exposed to the kairomone and experienced this factor themselves (Fig. 2, variant FF—red “x”). Statistically significant downward migration was observed from the first to the fourth day of the experiment, also in individuals whose mothers were not exposed to the kairomone but experienced the factor themselves (Fig. 2, variant CF—orange “x”). *Daphnia* that were not exposed to the kairomone, but whose mothers were, migrated upwards in response to water exchange. This response was statistically significant on the fifth day of the experiment. Animals exposed to kairomone whose mothers were not exposed to this factor resided deepest throughout the experiment (Fig. 2, CF). They remained significantly deeper than individuals exposed to kairomone whose mothers were also exposed to this factor (FF series), and from the second day onwards, they remained significantly deeper than control animals (Fig. 2, orange asterisks). Kairomone-exposed individuals whose mothers were also exposed to this factor (FF series) stayed deeper than the control group from the third day of the experiment onwards (Fig. 2, red asterisks). *Daphnia* not actually exposed to the kairomone, but whose mothers were, remained significantly shallower than control animals on the fourth and fifth days of the experiment (Fig. 2, yellow asterisks). Figure 2 illustrates two trends observed in animals exposed to a kairomone. The change in selected depth following the daily refreshment of medium and kairomone of those *Daphnia* whose mothers were exposed to this factor increased throughout the experiment. Also, the chosen depth of those *Daphnia* whose mothers were not exposed to kairomone consequently increased with time.

**Table I:** The effect of all tested factors on the depth residence of Daphnia (nested Linear Mixed Effects Model—LMM)

	Num df	Den df	*F*	*P*
**DAY-age of animals**	**5**	**14**	**11.95**	**0.0001**
**DE-direct effect of kairomone**	**1**	**1 682**	**606.32**	**<0.0001**
**ME-maternal effect of kairomone**	**1**	**1 681**	**185.20**	**<0.0001**
**FM-effect of refreshing medium**	**1**	**1 679**	**74.59**	**<0.0001**
**DAYxDE**	**5**	**1 691**	**31.73**	**<0.0001**
**DAYxME**	**5**	**1 686**	**2.64**	**0.0217**
**DExME**	**1**	**1 679**	**49.47**	**<0.0001**
DAYxFM	5	1 679	1.47	0.1958
**TExFM**	**1**	**1 679**	**66.05**	**<0.0001**
NExFM	1	1 678	0.10	0.7541
DAYxDExME	5	1 681	1.61	0.1546
**DAYxDExFM**	**5**	**1 679**	**2.44**	**0.0326**
DAYxMExFM	5	1 678	2.13	0.0590
**DExMExFM**	**1**	**1 678**	**13.41**	**0.0003**
**DAYxDExMExFM**	**5**	**1 679**	**2.6612**	**0.0210**

**Fig. 3:**
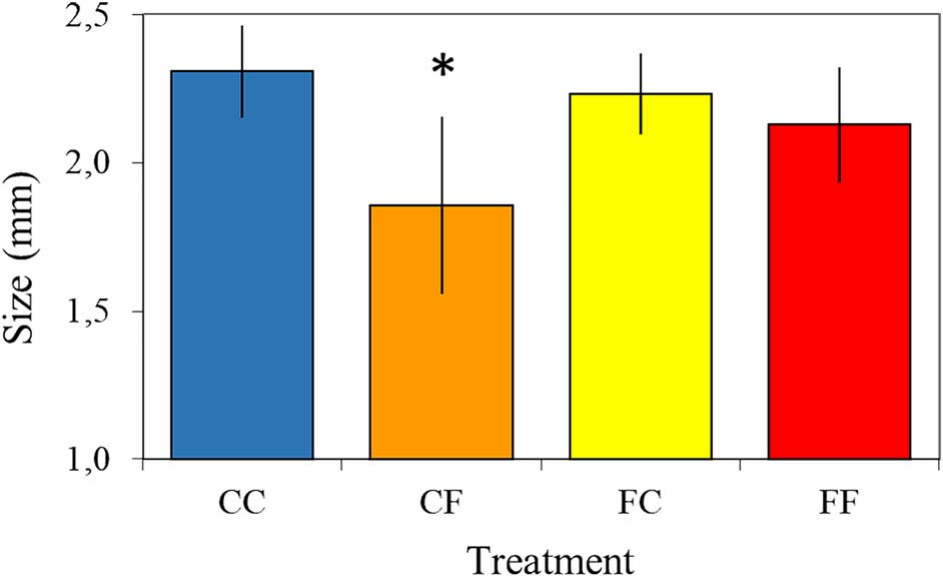
Size of *Daphnia* at Day 6 of the experiment, mean +/− 1 SD; asterisks indicate significant differences as compared to control animal (CC)—Mann–Whitney test with Bonferroni correction, *P* < 0.05.

The size of *Daphnia* was significantly affected by experimental conditions (*H*_(3, N = 62)_ = 20.85; *P* < 0.0001). At the end of the experiment, those exposed to the presence of a predator were significantly smaller than the control group (see Fig. 3). However, within this group, those with mothers exposed to fish kairomone were significantly larger (Mann–Whitney test, *P* < 0.05).

## DISCUSSION

Neither of the two hypotheses proposed in our study was supported. While ME can significantly modify *Daphnia*'s behavioral responses to threat, the direction of these modifications differed from what was expected. Offspring from mothers not exposed to kairomone (CF) exhibited a stronger, more costly response to the presence of kairomone, characterized by permanent residence in deeper, cooler waters. The depth at which they resided clearly increased with time (i.e. with individual age and body size) and with associated increasing predation risk (as it was earlier demonstrated by e.g. Loose, 1993; Hansson and Hylander, 2009). This strategy resulted in smaller body sizes at the end of the experiment compared to individuals from other treatments. Conversely, individuals from mothers exposed to kairomone exhibited the highest migration amplitude in response to addition of medium with fresh kairomone, which increased with experimental time and thus, individual age. They employed a more cost-saving strategy, resulting in smaller reduction in body size. Another unexpected observation was for individuals not exposed to kairomones, but whose mothers were. During the second part of the experiment, these individuals exhibited reverse migration in response to media exchange and the supply of fresh food. The animals' average depth decreased due to their upward migration, most probably in search of food and warmer water, which are factors that determine rapid growth.

Both behavior and size responses of *Daphnia* whose mothers were exposed to kairomones suggest their different prioritization in resolving trade-offs related to maximizing growth rate while minimizing the risk of death. The underlying cause of the observed phenomenon is, however, far from being fully recognized. The simplest explanations are that these animals either perceive their susceptibility to fish predation as lower, or that they have a greater demand for resources. It is difficult to find justification for the former, unless we assume that the mother's success in confronting the threat (as measured by their survival and successful reproduction) may indicate that it is not as serious as environmental signals would suggest. The latter can be explained in the light of optimization strategy implemented over several generations. The energetic costs associated with activating defense mechanisms in the first generation would motivate animals to reduce these costs in the next generation, i.e. to use strategies that potentially provide less security but a higher growth rate. This concept is consistent with the *Predation Risk Allocation Hypothesis*, which was formulated by (Lima and Bednekoff 1999). According to this hypothesis, animals should exhibit the strongest anti-predator behaviors in high-risk situations that are brief and rare, and frequent exposure to stress should stimulate more courageous behaviors (e.g. Fraser and Gilliam, 1987). Applying this to the ME, maternal exposure to threat may indeed encourage offspring to invest economically in defense mechanisms. This was confirmed by the results of Donelan and Trussell (2015), who demonstrated that the offspring of snails *Nucella lapillus* exposed to predators exhibit greater boldness than those of unexposed individuals.

The vertical distribution and migrations of *Daphnia* constitute a compromise between the need to remain in the warm, food-rich waters near the surface and the need to avoid predation by visually hunting fish (Dawidowicz and Loose, 1992). Therefore, it is not surprising that larger individuals (i.e. older ones, in the later stages of the experiment) that are more vulnerable to fish predation stay deeper or perform migrations of a larger amplitude (Fig. 2).

Our results clearly demonstrated that the assumption of rapid kairomone degradation in the experimental setup was correct. *Daphnia* exposed to the kairomone for two generations (the FF group), which exhibited the strongest migration in response to the kairomone, typically returned to the depth chosen by unexposed individuals before the medium was replaced the following day. This suggests that the kairomone degraded to concentrations below *Daphnia*'s behavioral response threshold and that no memory of the threat was developed to maintain defense with no signal of the danger. However, the first generation of *Daphnia* exposed to the kairomone (the CF group) apparently preserved such memory, determining their permanent presence in deeper water layers.

In typical temperate lakes, fish activity in the pelagic zone occurs in a circadian rhythm throughout the summer, with cyprinids migrating from the littoral zone to the center of the lake in the evening, and whitefish or smelt migrating upwards from the hypolimnion (Gliwicz and Jachner, 1992; Gliwicz and Dawidowicz, 2001). In such a situation, DVM would be induced anew each day. However, given the relatively long reaction time, migration response could be delayed and, as mentioned in the introduction, be ineffective. The observed mechanism more closely matches the pattern of fish pressure observed in the Binnensee, the native reservoir of the studied *Daphnia* genotype, where fish appear periodically. In case of periodic fish appearances, a costly and safety-oriented response by the first generation of *Daphnia* in the face of a threat that may soon disappear seems adaptive, as well as a strategy aimed at compensating for the costs of the implemented mechanisms in subsequent generations.

Since genetic variation can strongly influence *Daphnia* responses to predator cues (Pijanowska *et al.,* 1993), without considering potential genotype-specific differences, it is difficult to determine whether the observed responses reflect a general or clone-specific pattern of ME. Our results cover only a snapshot of the whole realm of flexible responses of *Daphnia* to environmental variability and therefore we do not aspire to perceive our conclusions as universal. Moreover, we believe that such a universal pattern does not exist. Multiplying the number of investigated clones and habitats, one will certainly approach to the more complex vision, without however the possibility of seizing the general algorithm.

Regardless of the ecological implications of the observed mechanisms, the rapid degradation of the kairomone and following reversal of the behavioral response to its presence within a few hours are of methodological importance. When testing *Daphnia*'s behavioral responses to kairomones in batch or flow-through cultures, fresh kairomone is typically introduced into the system once per day. If so, results obtained at different times before or after replacing the medium may differ significantly, leading to different conclusions. Furthermore, the exposure of the tested individuals to the kairomone is pulsed rather than continuous. This is worth consideration when planning experiments and interpreting the results. Our study provides the further evidence of the complexity of reactions in the fear landscape. The optimization of the organism's defensive reactions is based on the perception of many interacting factors, including, information from the mother indicating their potential duration.

## CONCLUSIONS

- The ME is involved in the induction of behavioral variation in *Daphnia*—the fear experienced by the mother discourages their offspring from incurring the costs of DVM.
- The process of optimizing defense mechanisms probably involves decisions being made across several generations, including compensation for the previous generation's defense costs.
- Indeed, fish kairomone degrades in less than 24 hours, which could result in the defense mechanism becoming inactive during this time. This is an important factor to consider when planning and interpreting experiments.

## Data Availability

Data is available upon reasonable request to the corresponding author.

## Notes

Corresponding editor: Lars-Anders Hansson
